# Childhood Hypopigmented Mycosis Fungoides: A Rare Diagnosis

**DOI:** 10.1155/2016/8564389

**Published:** 2016-11-29

**Authors:** Cláudia Patraquim, Maria Miguel Gomes, Carla Garcez, Filipa Leite, Tereza Oliva, António Santos, Armando Pinto

**Affiliations:** ^1^Pediatrics Department, Hospital de Braga, Sete Fontes, São Victor, 4710-243 Braga, Portugal; ^2^Pediatrics Department, Portuguese Oncology Institute of Porto Francisco Gentil, 4200-072 Porto, Portugal; ^3^Dermatology Department, Portuguese Oncology Institute of Porto Francisco Gentil, 4200-072 Porto, Portugal

## Abstract

Primary cutaneous lymphomas (PCL) are rare in pediatrics. Mycosis fungoides (MF) is the most frequent PCL diagnosed in childhood. There are various clinical variants of MF, including the hypopigmented MF (HMF). We present a 5-year-old boy with an 18-month history of progressive, generalized, nonpruritic hypopigmented lesions with central lacy erythema. He had no improvement with emollients. Skin biopsy showed typical features of HMF. He was treated with topical corticosteroids and tacrolimus and narrow-band ultraviolet B (NBUVB) phototherapy, with good response. HMF may mimic multiple skin disorders. Unusual hypopigmented skin lesions should be biopsied. Though phototherapy is effective, recurrence is common.

## 1. Introduction

Primary cutaneous lymphomas (PCL) are a heterogeneous group of T- and B-cell lymphomas that are uncommon in children and adolescents [1, 2].

Mycosis fungoides (MF), the most frequent subtype of cutaneous T-cell lymphoma (CTCL), [3, 4] is classified as an indolent lymphoma, according to the WHO-EORTC classification of PCL [2, 5].

Although MF is typically present in older ages (medium age at diagnosis of 55–60 years, with a 2 : 1 male to female predominance) [1, 2, 4, 5] it often represents the most diagnosed PCL in childhood [2].

There are several distinct clinical forms of MF [3]. The hypopigmented MF (HMF) is a rare variant which occurs more often in dark-skinned individuals and Asians, especially in the first or second decade of life, and commonly shows a T-suppressor CD8+ phenotype [2, 3, 6, 7].

Misdiagnosis of HMF as any of a range of benign skin disorders is frequent because it can have clinical and histological resemblances with multiple inflammatory dermatoses [3, 8]. As a consequence, the diagnosis of HMF in childhood is commonly delayed [2, 3].

This case aims to raise awareness regarding the importance of clinical suspicion for MF in patients, mainly children, with persistent, progressive, and/or unusual hypopigmented skin lesions.

## 2. Case Description

**Figure 1:**
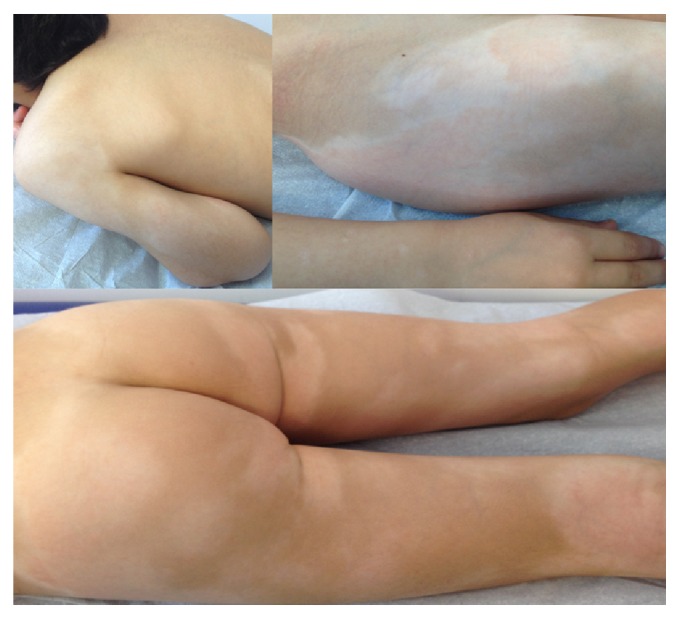
Patient presentation: generalized hypopigmented patches with central lacy erythema.

The patient is a 5-year-old Caucasian boy with an 18-month history of progressive, generalized, nonpruritic hypopigmented lesions, with central lacy erythema and hypopigmented halo, associated with few erythematous papules, within normal overlying skin. The largest lesion was located in the iliac crest (Figure 1).

The patient's past medical and family history were irrelevant, with no evidence of recent infections, atopy, other inflammatory dermatosis, or relevant environmental exposure.

**Figure 2:**
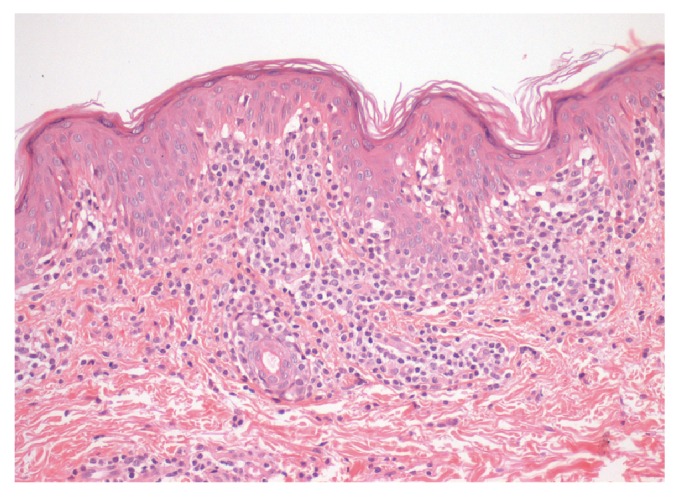
Dermatopathology: papillary dermal interstitial infiltrate of lymphocytes with mild atypia and epidermotropism.

He was firstly diagnosed with a benign skin condition and prescribed emollients, with no improvement. However, given the persistence and progression of the skin lesions, the patient was submitted to biopsy from a hypopigmented patch that showed typical features of hypopigmented MF (papillary dermal interstitial infiltrate of lymphocytes with mild atypia and epidermotropism; Figure 2). Immunophenotyping showed positivity of atypical lymphoid cells for CD2, CD3 (with decreased expression intensity), CD5, and CD8 and an absence of expression of CD20, CD4, and CD30.

On physical examination he had infracentimetric cervical, axillary, and inguinal lymph nodes and no evidence of organomegaly.

Laboratory tests (complete blood count with differential, biochemistry including renal and hepatic function, hemostasis, immunoglobulins, peripheral blood immunophenotyping, and infectious serologies) were unremarkable. Positron emission tomography (PET) scanning was suggestive of metabolically active lymphoproliferative disease with cervical lymph node involvement. Cervical lymph node excisional biopsy did not show involvement by lymphoid neoplasia. Bone marrow aspirate and biopsy were likewise normal.

Therefore, staging tests revealed localized cutaneous disease and the patient was diagnosed with HMF, stage Ib (T2N0M0B0). He initiated treatment with topical corticosteroid 3 times weekly and tacrolimus 2 times weekly and narrow-band ultraviolet B (NBUVB) phototherapy sessions, 2-3 times weekly, with good response.

After the patient began phototherapy sessions, he reported pruritus which significantly improved with continued treatment. Resolution of the central lacy erythema became apparent after a few sessions.

A significant improvement of hypopigmented patches and an absence of new lesions were observed at his last visit (approximately 1 year after diagnosis). Thirty-two NBUVB phototherapy sessions were performed so far, in two different periods of time (cumulative dose 15.8 J/cm^2^), combined with topical mometasone cream 2 times a week in residual macular lesions.

## 3. Discussion

The incidence of MF is overall low, yet it represents the most frequent PCL diagnosed in both the pediatric age group and adults [9]. Its prevalence is predominantly higher in adults; however the hypopigmented variant is comparatively common among children [2, 4, 9, 10]. Hypopigmented skin lesions are overrepresented in juvenile-onset MF, as reported in the studies from Boulos et al. and Hodak and coworkers, in which 53% of 34 patients and 58% of 50 children/adolescents, respectively, had HMF [4, 10].

The presence of hypopigmentation is considered a marker of good prognosis, compared to the classical MF [2, 3].

Cutaneous expression of MF is divided in the following stages: early patch, plaque, tumour, and an erythrodermic stage [2]. As an indolent lymphoma, MF usually has a slow progression, over years or even decades through the mentioned phases [2, 5]. In later stages of the disease, patients may also develop extracutaneous disease with involvement of lymph nodes and visceral organs [5]. Fortunately, this condition is exceedingly rare, particularly in children who generally present with early stage disease consisting of limited or widespread patch or plaque stage. Palpable lymph nodes are usually free of disease, and the majority of affected children are diagnosed with stage Ia, Ib, or IIa MF, which is in line with the present case [4, 9–13]. An analysis of 36 pediatric patients from Kuwait reported that patch stage disease was the most common clinical variant (75%) and most patients had stage Ib disease [11]. Scarlett Boulos and coworkers showed that 41% and 56% of their 34 cases of juvenile-onset MF (median age at diagnosis 14 years) were at stages Ia and Ib, respectively [4]. Likewise, other studies reported identical findings [12, 13].

Distinctive skin manifestations of HMF include hypopigmented-to-achromic lesions, of variable sizes, which are often confined to the trunk, buttocks, pelvic girdle, and lower limbs, albeit any body surface may be affected. Pruritus of variable intensity may also be present [3].

Differential diagnosis is vast. The prevalence of HMF is certainly underestimated because it may mimic several benign skin conditions such as atopic dermatitis, pityriasis alba, vitiligo, postinflammatory hypopigmentation, pityriasis lichenoides chronica, and pityriasis versicolor, among others [2, 3, 6]. Hence, diagnosis must include clinicopathologic correlation [3]. Some of the most common alterations in HMF skin biopsy analysis are prominent epidermotropism characterized by atypical CD8+ with convoluted nuclei, compared to the classical MF. Hypopigmentation is considered a good prognostic indicator because these CD8+ cells are involved in T helper 1-mediated immune responses and, subsequently, may prevent evolution to advanced stages of the disease (disease progression follows a shift from Th1 to Th2 response), even though they are malignant. Moreover, these cells can also contribute to the inhibition of melanogenesis [2, 3].

Treatment options for HMF comprise topical corticosteroids alone or in combination with NBUVB phototherapy sessions or with psoralen and ultraviolet A (PUVA) [2, 7]. A large and long-term retrospective study on pediatric HMF reported that 45.7% of patients significantly improved with phototherapy sessions; nevertheless 20% had recurrence of the disease after therapy was suspended and only 7% had complete remission. One patient progressed to plaque/tumor stage after being lost to follow-up for 5 years [7]. Laws and coworkers reported partial or complete response in 86% of patients after phototherapy with no disease progression during the period of study (median 43 months) [12].

Evidence is scarce regarding the comparison of efficacy of NBUVB phototherapy with other forms of light therapy [9]. Heng and coworkers showed that PUVA is now used much less often than NBUVB phototherapy, since it is considered to be safer [13]. Similarly, a retrospective study from Koh and Chong concluded that NBUVB phototherapy is an effective and safe treatment for early stage MF in children, yet recurrence is frequent. Therefore, long-term follow-up is essential for all patients with MF [9].

The use of topical tacrolimus, a calcineurin inhibitor, is controversial. It has been associated with a theoretical but still unclear risk for secondary malignancies including CTCL [14, 15]. Rallis and coworkers described a case of a 29-year-old man with patch type MF that was successfully treated with tacrolimus ointment [15]. Our patient was treated with topical tacrolimus 2 times weekly for a period of 2 months, as indicated and prescribed in the dermatology appointments.

In conclusion, HMF is an uncommon and challenging disease which resembles frequent skin disorders; therefore suspicious skin lesions should be biopsied to avoid diagnostic delays, especially in children. Finally, topical corticosteroids and NBUVB phototherapy are safe and effective despite relapses being common.

## Abbreviations

CTCL: Cutaneous T-cell lymphoma
HMF: Hypopigmented mycosis fungoides
MF: Mycosis fungoides
NBUVB: Narrow-band ultraviolet B
PCL: Primary cutaneous lymphomas
PET: Positron emission tomography
PUVA: Psoralen and ultraviolet A.
